# Efficacy, safety, and anti-inflammatory properties of the switch to a doravirine-based regimen among antiretroviral-experienced elderly people living with HIV-1: the DORAGE cohort

**DOI:** 10.1128/aac.00815-24

**Published:** 2025-02-18

**Authors:** Alessandro Lazzaro, Gregorio Egidio Recchia, Federica Alessi, Letizia Santinelli, Luigi Battistini, Julieta Morcos, Francesco Romano, Ginevra Bugani, Luca Maddaloni, Sara Caruso, Marta D'Amico, Ivano Mezzaroma, Mario Falciano, Caterina Fimiani, Germana Sfara, Maria Gemma Leone, Ombretta Turriziani, Claudio Maria Mastroianni, Gabriella d’Ettorre

**Affiliations:** 1 Department of Public Health and Infectious Diseases, Sapienza University of Rome 119653 , Rome, Italy; 2 Department of Translational and Precision Medicine, Sapienza University of Rome 117537 , Rome, Italy; 3 Department of Molecular Medicine, Sapienza University of Rome 315098 , Rome, Italy; IrsiCaixa Institut de Recerca de la Sida, Barcelona, Spain

**Keywords:** doravirine, HIV-1, ART, NNRTIs, inflammation, metabolic profile

## Abstract

Doravirine (DOR) is a novel antiretroviral agent with a favorable resistance profile and high tolerability. However, evidence is limited on DOR among elderly people living with HIV (PLWH) and whether it might modulate chronic inflammation. We aimed to investigate the efficacy, safety, and tolerability of DOR as a switching strategy among elderly PLWH and its impact on chronic inflammation in a real-life setting. We recruited a cohort of ART-experienced PLWH undergoing a therapeutic switch to a DOR-based regimen under virologic control (defined as HIV-RNA <200 copies/mL), regardless of the previous ART regimen. The primary objective was the evaluation of the rate of virologic control at 48 weeks post-switch. Secondary objectives included analyzing immune and metabolic outcomes. Plasmatic hs-CRP, IL-6, and D-dimer levels were measured as chronic inflammation markers. Overall, 150 PLWH were screened, and 147 were enrolled into the study. A total of 134 PLWH completed the follow-up. The rate of virological control was 96.1% (122/134; *CIs*: 91.0%–98.7%) in the per-protocol analysis. After 48 weeks from the switch, we recorded significant reductions in serum fasting glycemia (*P* 0.019), triglycerides (*P* 0.024), and total cholesterol/HDL ratio (*P* 0.017); no clinically significant differences were detected in the body weight and BMI, as long as in immune, hepatic, and renal profiles. A significant reduction in IL-6 (*P* 0.019) and hs-CRP (*P* 0.019) was observed. DOR is an effective and safe treatment choice for elderly PLWH. The intriguing modulatory effect of DOR-based regimens on chronic systemic inflammation deserves further investigation.

## INTRODUCTION

The advent of combined antiretroviral therapy (ART) dramatically changed the natural course of human immunodeficiency virus type 1 (HIV-1) infection and improved patient survival (1). Over 20 years after the approval of the first non-nucleoside reverse transcriptase inhibitor (NNRTI) by US Food and Drug Administration (FDA), nevirapine, in 1996, the NNRTI class still represents a cornerstone of combination ART. Nowadays, NNRTIs find application in both first- and second-line strategies, including triple and dual drug regimens, as well as single-tablet and/or long-acting formulations. Moreover, NNRTIs remain a cornerstone of ART in resource-limited countries, despite the recommendations by the World Health Organization (WHO) (2).

Doravirine (DOR) is the last pyridonone NNRTI approved by the US FDA in 2018 and licensed for both naïve and ART-experienced people living with HIV (PLWH) (3). Due to its high selectivity for the viral reverse transcriptase (does not bind eucaryotic polymerases) (4) and its unique pharmacokinetic profile, DOR represents a novel and valuable weapon against HIV-1 strains carrying resistance-associated mutations (RAMs) to first (K103N) and/or second (E138K, Y181C) generation of NNRTIs (5). Its favorable pharmacokinetic features (lower protein binding that leads to higher trough concentrations compared to other NNRTIs, an elimination half-life of around 15 hours, median time to peak plasma concentrations of 1–4 hours, and a time to steady-state concentration of 7 days), along with its relative stability regardless of sex, age, race, or hepatic impairment, contribute to both the DOR’s high virologic efficacy and its tolerability (6). Indeed, in contrast to first-generation NNRTIs (nevirapine and efavirenz), which are associated to liver toxicity, second-generation NNRTIs [etravirine, rilpivirine (RPV), and DOR] are not (7). Additionally, DOR is less likely to cause cardiotoxicity related to off-target effects on cardiac ion channels, as well as neuropsychiatric adverse events, unlike previous NNRTIs (8).

DOR is available both as a single agent and co-formulated with two nucleos(t)ide reverse transcriptase inhibitors, namely, lamivudine (3TC) and tenofovir disoproxil fumarate (TDF), as part of a single-tablet regimen (STR). Its favorable resistance profile, along with its high tolerability and low potential for drug–drug interactions, makes DOR a viable NNRTI option for both NNRTI-naïve and NNRTI-experienced PLWH (8). Pivotal randomized clinical trials have demonstrated that DOR is a safe choice among PLWH treated with boosted protease inhibitors (PIs) for a switching regimen (9), showing a favorable lipidic profile. However, whether the improvement in serum fasting lipid levels is associated with a reduction in chronic systemic inflammation remains unknown. Furthermore, real-life data, especially among key population such as females and elderly, remain limited.

In this study, we aimed to assess the virologic efficacy, safety, and tolerability of the DOR-based regimens in an observational cohort of elderly PLWH.

## MATERIALS AND METHODS

### Study design

The DORAGE cohort is a single-arm, non-controlled, prospective switch cohort, describing data from PLWH, followed as out-patients at the Department of Tropical and Infectious Diseases, who switched their current ART to a DOR-based regimen, independently from the previous ART taken. The primary objective was to assess the virologic efficacy of DOR among PLWH older than 50 in a real-life setting; the primary endpoint was the rate of subjects with HIV-RNA <50 copies/mL at week 48 (w48), assessed using the US Food and Drug Administration (FDA)’s Snapshot algorithm (10). Secondary objectives included virologic efficacy assessment at week 24 (w24); prevalence of pre-existing RAMs to NNRTIs; immune profile changes from baseline (BL) to w48 (secondary endpoint: CD4^+^ T cells, CD8^+^ T cells, and CD4^+^/CD8^+^ ratio); metabolic profile changes from BL to w48 [secondary endpoints: fasting serum total (TC), low-density lipoprotein (LDL), high-density lipoprotein (HDL) cholesterol, TC/HDL ratio, body weight, BMI, creatinine, and estimated glomerular filtration rate (eGFR, as estimated with the eGFR–EPI–CKD–2021 formula)] (11); inflammatory profile changes from BL to w48 [secondary endpoints: serum interleukin (IL)−6, high-sensitivity C-reactive protein (hs-CRP), and D-dimer); tolerability profile of DOR along the study (secondary endpoint: proportion of serious adverse events reported from BL to w48).

Inclusion criteria included being aged ≥50 years and having ART-experienced HIV-1 infection under virologic control, defined as virologic suppression (HIV-RNA ≤50 copies/mL for at least 6 months prior to enrollment) or as stable, persistent low-level viremia (HIV-RNA ≥50 but <200 copies/mL). Exclusion criteria (either present at the moment of the switch or during the study period) included a history or evidence of major RAMs to DOR (12) using a chart review and GRT (when available); autoimmune and/or neoplastic disease; acute hepatitis, chronic liver disease, or liver cirrhosis; severe or end-stage chronic kidney disease; concomitant immunosuppressive therapies; risk for potential drug–drug interactions between DOR and current medications taken by participants, as reported by the University of Liverpool dedicated website (13); pregnancy and/or breast-feeding. The onset of an exclusion criterion during the study period would result in withdrawal from the study.

The following information was extracted from medical records and collected into the cohort database: age (years); sex; comorbidities (type 2 diabetes, hypertension, and dyslipidemia); body weight (Kg); body mass index (BMI, Kg/m^2^); creatinine (mg/dL); eGFR (mL/min/1.73 m^2^); TC, LDL, and HDL (mg/dL); TC/HDL ratio; HIV-RNA (copies/mL); CD4^+^ T and CD8^+^ T lymphocytes (cells/µL) and CD4^+^/CD8^+^ ratio; all available genotypic resistance tests (GRTs); and clinical assessment of drug side effects. Adverse events (AEs) were classified as mild/moderate, severe, or life-threatening, according to the Division of AIDS Classification (14). AEs were considered unrelated to DOR, possibly related or related, according to the physician’s evaluation as part of routine follow-up. Tolerability was measured by the number of treatment interruptions due to drug-related adverse events (15).

In order to examine the prevalence of pre-switch NNRTI-associated RAMs in the enrolled subjects and provide real-life data of the DOR resistance profile, we retrospectively collected all available historical GRTs to build a cumulative genotypic resistance profile. We assessed the pre-switch drug resistance mutation score for DOR according to the Stanford algorithm (12). The virologic outcome at w48 was retrospectively analyzed to observe the impact of RAMs on the regimen efficacy. Participants with confirmed virologic failure (VF), as defined by HIV-RNA ≥200 copies/mL, underwent GRT. RAMs were considered treatment-emergent when detected during the observation period in participants without historical genotypic data or if absent in historical data for participants with an available GRT at BL.

### Enzyme-linked immunosorbent assay

Plasma samples were collected from fresh peripheral blood samples obtained by venipuncture in Vacutainer tubes containing EDTA (BD Biosciences). Plasma levels of IL-6 (RayBiotech Life, USA), hs-CRP (ELK Biotechnology, USA), and D-dimer (Abcam, UK) were detected by enzyme-linked immunosorbent assay (ELISA) kits, following the manufacturer’s instructions. The assay was based on an indirect assay with an antigen–antiserum bound and a conjugate antibody using a 3,3′,5, 5′-tetramethylbenzidine (TMB) as a substrate for the detection. The reactions were monitored at 450 nm. Quantification was achieved by comparing their absorbance with a reference curve (prepared with known standard concentrations). The calculated minimal detectable dose (MDD) was 3 pg/mL for IL-6, 26.1 pg/mL for hs-CRP, and 2.36 ng/mL for D-dimer.

### Statistical analysis

BL characteristics of enrolled patients were considered as median values and interquartile range (IQR) or simple frequencies (n) and proportions (percentages, %), depending on whether the variable was continuous or categorical, respectively. The normality of variable distribution was assessed by the Shapiro–Wilk statistics. The Clopper–Pearson exact method was used to calculate 95% confidence intervals (*CIs*) for virologic data with both the intention-to-treat (missing as failure) and the per-protocol (missing as excluded) analyses. Changes from BL were assessed as the median of raw absolute difference (w24 value−BL value; w48 value−BL value) or the median of percentage relative difference ([w24−BL]/BL ×100; [w48−BL]/BL ×100). Paired longitudinal analyses were performed using the paired Wilcoxon test for continuous variables. All tests were two-sided, and a *P*-value of less than 0.05 was considered statistically significant, with false discovery rate (FDR) correction for multiple comparisons applied when appropriate. All data were analyzed using RStudio (Version 2022.12.0+353 Copyright© 2022 by Posit Software, PBC) and Microsoft Excel for Mac (Version 16.48).

## RESULTS

### Population features

Overall, 150 PLWH were screened, and at the end of the enrollment, 147 PLWH were included in the study. Thirteen participants discontinued the study due to personal preference, as switching from an STR to a multi--lets DOR-based ART impacted their adherence, prompting them to return to the previous regimen. A total of 134 PLWH completed the follow-up and are further described. Demographic, medical history, and treatment details are summarized in Table 1. Despite a predominance of men in the study population (101/134, 75.4%*—P* < 0.001), no clinically relevant differences by sex were observed at BL. The median CD4^+^ T cells at BL was 685 (56 – 895) cells/µL, with a median time on pre-switch ART regimen of 36 (24 – 48) months; about one-third of the population had a nadir <200 cells/µL at the time of HIV-1 diagnosis. The majority of participants (129, 96.3%) presented virologic suppression at BL, except for five participants (3.7%) showing low-level viremia. Almost half of the enrolled participants were affected by arterial systemic hypertension and/or dyslipidemia, and up to 20% suffered from type 2 diabetes.

**TABLE 1:** Demographics of the overall population and by sex

	Overall (*n* = 134)	Female (*n* = 33)	Male (*n* = 101)	*P*-value
Sex (female/male)	33/101	24 (60%)	75 (40%)	**<0.001**
Ethnicity (Caucasian)	121 (90.3%)	27 (81.8%)	94 (93.1%)	0.086
Age (years)	58 (54–65)	59 (55–64)	58 (54–65)	0.464
Type 2 diabetes (yes)	26 (19.4%)	7 (21.2%)	19 (18.8%)	0.805
Dyslipidemia (yes)	62 (46.3%)	18 (54.5%)	44 (43.6%)	0.421
Hypertension (yes)	64 (47.8%)	14 (42.4%)	50 (49.5%)	0.426
CD4^+^ T cells nadir (cells/µL)	150 (78–230)	170 (89–220)	130 (78–230)	0.768
CD4 T cell nadir <200 cells/µL (yes)	44 (32.8%)	9 (27.3%)	35 (34.7%)	0.524
CD4^+^ T cells at baseline (cells/µL)	685 (56–895)	754 (662–987)	672 (530–868)	**0.044**
Time since ART initiation (months)	216 (126–276)	192 (120–312)	216 (129–264)	0.639
Time on pre-switch ART (months)	36 (24–48)	36 (24–36)	36 (24–60)	0.172
HIV-RNA zenith (copies/mL)	78,000 (556,000–100,000)	74,000 (33,000–92,000)	87,000 (56,000–100,000)	0.068
HIV-RNA <50 copies/mL at baseline [*n* (%)]	129 (96.3%)	32 (17.2%)	97 (58.9%)	**0.002**
HIV-RNA 50–200 copies/mL at baseline [*n* (%)]	5 (3.7%)	1 (0.7%)	4 (2.9%)	-

^*a*^ *P*-values < 0.05 are highlighted in bold. ART: antiretroviral therapy.

Antiretroviral regimens used pre- and post-switch to the DOR-based regimens are depicted in Table 2. NRTIs were widely used before the switch (*n* = 116, 86.6%), with a major prevalence of tenofovir alafenamide fumarate (TAF) (*n* = 74, 55.2%). After the switch to a DOR-based ART, the DOR/3TC/TDF STR was the most prescribed regimen (*n* = 52, 38.8%), followed by DOR combined with emtricitabine (FTC)/TAF, dolutegravir (DTG), and raltegravir (RAL), in the order. DOR was combined with an integrase strand transfer inhibitor (INSTI) as a dual-drug regimen in 50 (37.3%) cases. One participant receiving RPV + darunavir/cobicistat/FTC/TAF at BL was switched from the RPV to DOR regimen and continued darunavir/cobicistat/FTC/TAF.

**TABLE 2:** Pre- and post-switch ART regimens^*a*^

Post-switch ART regimens		Pre-switch ART regimens			
		By class		By drug	
DOR/3TC/TDF	52 (38.8%)	INSTI + 2NRTI	50 (37.3%)	BIC/FTC/TAF	5 (3.7%)
DOR + FTC/TAF	31 (23.1%)	INSTI + PI	9 (6.7%)	DRV/COBI/FTC/TAF	25 (18.7%)
DOR + DTG	39 (29.1%)	INSTI + 1NRTI	7 (5.2%)	DRV/COBI + ABC/3TC	1 (0.7%)
DOR + RAL	11 (8.2%)	INSTI + NNRTI	6 (4.5%)	DTG/3TC	5 (3.7%)
DOR + DRV/COBI/FTC/TAF	1 (0.7%)	NNRTI + 2NRTI	34 (25.4%)	DTG/ABC/3TC	7 (5.2%)
		PI + 2NRTI	26 (19.4%)	DTG/RPV	3 (2.2%)
		INSTI + NNRTI + MVC	1 (0.7%)	DTG + DRV/COBI	1 (0.7%)
		NNRTI + PI + 2NRTI	1 (0.7%)	DTG + FTC/TAF	5 (3.7%)
				DTG + FTC/TDF	2 (1.5%)
				EFV/FTC/TDF	5 (3.7%)
				EVG/COBI/FTC/TAF	2 (1.5%)
				NVP + ABC/3TC	1 (0.7%)
				NVP + FTC/TAF	2 (1.5%)
				NVP + FTC/TDF	6 (4.5%)
				RAL + 3TC	2 (1.5%)
				RAL + ABC/3TC	7 (5.2%)
				RAL + DRV/COBI	8 (6.0%)
				RAL + FTC/TAF	14 (10.4%)

^*a*^ 3TC: lamivudine; ABC: abacavir; ART: antiretroviral therapy; BIC: bictegravir; COBI: cobicistat; DRV: darunavir; DTG: dolutegravir; EFV: efavirenz; EVG: elvitegravir; FTC: emtricitabine; INSTI: integrase strand transfer inhibitors; MVC: maraviroc; NNRTI: non-nucleoside reverse transcriptase inhibitors; NRTI: nucleos(t)ide reverse transcriptase inhibitors; NVP: nevirapine; PI: protease inhibitors; TDF: tenofovir disoproxil fumarate; TAF: tenofovir alafenamide fumarate; RAL: raltegravir; RPV: rilpivirine.

### Virologic results

**Fig 1:**
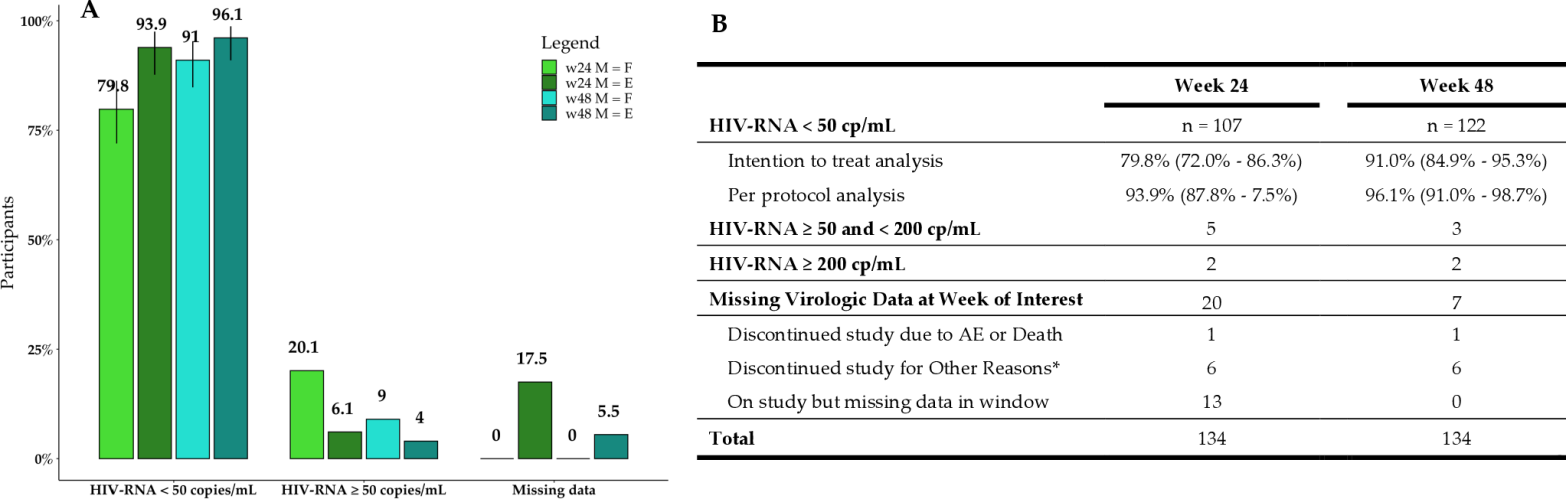
(**A**) Virologic control (HIV-RNA <50 copies/mL), virologic failure (HIV-RNA ≥50 copies/mL), and missing virologic data at week 24 (w24) and week 48 (w48). M = F : missing equal failure – intention-to-treat analysis; M = E : missing equal excluded – per-protocol analysis. The figure shows bars for both the intention-to-treat analysis considering missing as failure (w24 in pale green and w48 in pale blue), and the per-protocol analysis treating missing as excluded (w24 in dark green and w48 in dark blue). (**B**) Virologic outcomes at w48 and w96 according to FDA guidelines; confidence intervals are shown in parentheses. *Other reasons include subjects who discontinued study drugs due to the investigator’s discretion, subject decision, loss to follow-up, noncompliance with the study drug, protocol violation, and pregnancy.

Virologic data were evaluated according to the FDA snapshot algorithm, using HIV-RNA results available at the visits of interest (11). Percentages of virologic response and failure, as well as percentages of missing virologic data for both w24 and w48, are shown in Fig. 1A.

At w24, seven participants showed HIV-RNA >50 copies/mL, and virologic results were missing for 20 participants: out of them, one participant had died, 13 returned at w48 and completed the follow-up, while six dropped out for loss to follow-up. In the intention-to-treat analysis, at w24, a rate of virologic control of 79.8% was reported (107/134, *CIs*: 72.0%–86.3%). Nonetheless, in the per-protocol analysis, the rate of virologic control was 93.9% (107/114, *CIs*: 87.8%–97.5%). Out of seven participants with HIV-RNA >50 copies/mL at w24, five showed HIV-RNA <200 copies/mL and were maintained in the current ART according to EACS guidelines (3) and followed-up to w48. The two participants with HIV-RNA >200 copies/mL reported self-discontinuation of the study drug due to logistic issues: one returned to HIV-RNA <50 copies/mL at w48, while the other had persistent low-level viremia at the same time point, with a decline in HIV-RNA levels from 396 copies/mL to 175 copies/mL. Participants experiencing virologic failure underwent a medical interview, and a blood sample was collected to perform a GRT. However, no treatment-emergent RAMs were detected.

At w48, virologic data were available for 127/134 participants, with 122 PLWH showing HIV-RNA <50 copies/mL and five HIV-RNA >50 copies/mL. In the intention-to-treat analysis, a rate of virologic control of 91.0% at was reported (122/134, *CIs*: 84.9%–95.3%); while, in the per-protocol analysis, the rate of virologic control was 96.1% (122/127, *CIs*: 81.0%–98.7%). Among the five participants with HIV-RNA >50 copies/mL at w48, three had HIV-RNA <200 copies/mL (two of them were known to experience low-level viremia in their clinical history). The two participants experiencing virologic failure (namely, 222 copies/mL and 265 copies/mL) underwent GRT, but again, no treatment-emergent RAMs were detected (Fig. 1B).

### Pretreatment NNRTI-RAM prevalence and virologic performance of DOR-based regimens

Among the 134 participants, at least one GRT was available at BL in 88 cases. The prevalence of individual pretreatment NNRTI-RAMs is described in Table 3, while the patterns of RT-RAMs found among the nine PLWH with NNRTI-RAMs at BL are shown in Table 4. Nine (10.2% of 88) participants had at least one NNRTI-associated RAM at BL. All of them showed HIV-RNA <50 copies/mL after 48 weeks of observation.

**TABLE 3:** Frequencies of pre-treatment RAMs to NNRTIs before the switch, DRM score to DOR, and interpretation of the susceptibility pattern according to the Stanford algorithm (12)

RAM	Frequency	DRM score to DOR	Interpretation
V90I	3 (3.4%)	0	S
A98G	3 (3.4%)	15	LL-R
V106I	2 (2.3%)	10	PLL-R
V179D	1 (1.1%)	0	S
Y181C	1 (1.1%)	10	PLL-R

**TABLE 4:** Patterns of pre-treatment RT-RAMs^*a*^

Subjects	NNRTI-RAMs	Other RT-RAMs	Cumulative DRM score to DOR	Interpretation	Regimen assigned after the switch
#1	V90I	-^*b*^	0	S	DOR + F/TAF
#2	V90I	T215S	0	S	DOR/3TC/TDF
#3	V106I	-	10	PLL-R	DOR + DTG
#4	V106I	-	10	PLL-R	DOR + F/TAF
#5	V179D	-	0	S	DOR + DTG
#6	Y181C	M184V	10	PLL-R	DOR + DRV/c/F/TAF
#7	V90I + A98G	T215D	15	LL-R	DOR/3TC/TDF
#8	A98G	K70R + M184V	15	LL-R	DOR/3TC/TDF
#9	A98G	M41L + T69D +K70R +M184V +T215V +K219Q	15	LL-R	DOR + DTG

^*a*^ Patterns of pre-treatment RT-RAMs found among the 11 PLWH with NNRTI-RAMs, their cumulative DRM score to DOR and its relative interpretation, and DOR-based ART regimen after the switch. ART: antiretroviral therapy; 3TC: lamivudine; c: cobicistat; DOR: doravirine; DRM: drug resistance mutation; DRV: darunavir; DTG: dolutegravir; F: emtricitabine; HL-R: high-level resistance; I-R: intermediate resistance; LL-R: low-level resistance; PLL-R: potential low-level resistance; RAM: resistance-associated mutation; S: susceptible; TAF: tenofovir alafenamide fumarate; TDF: tenofovir disoproxil fumarate; RAL: raltegravir.

^*b*^ -, absence of other RT-RAMs.

### Immunologic profile

No significant changes were observed among the CD4^+^ and CD8^+^ T-cell subsets or the CD4^+^/CD8^+^ ratio between BL and both w24 and w48, as shown in Table 5.

**TABLE 5:** Immunological and metabolic changes between baseline week 24 and week 48^*a*^

Overall cohort (*n* = 134)	Median (IQR)			w24 vs BL			w48 vs BL		
	BL	w24	w48	Absolute difference	MRD %	*P*	Absolute difference	MRD %	*P*
Body weight (Kg)	76.0 (62.2–85)	76.0 (65–85)	76.0 (64.7–85)	0 (−1 to 0)	0 (−1.54 to 0)	0.476	0 (−2 to 0)	0 (−3.12 to 0)	**0.002**
BMI (Kg/m2)	25.9 (22.8–28.9)	25.6 (22.65–29.40)	25.5 (22.5–28.8)	0 (−0.38 to 0.1)	0 (−1.27 to 0.45)	0.651	0 (−0.8 to 0)	0 (−2.91 to 0)	**0.019**
CD4 + T Lymphocytes (cells/µL)	685 (560–895)	703 (508–914)	746 (613–949)	−6 (−100 to 107)	−1.1 (−13.29 to 18.07)	0.651	46 (−61 to 138)	6.4 (−8.39 to 22.04)	0.510
CD8 + T Lymphocytes (cells/µL)	833 (632–1,100)	842 (608–1,151)	887 (650–1,156)	−11 (−121 to 176)	−2.62 (−15.36 to 26.46)	0.651	38 (−64 to 181)	4.65 (−8.92 to 25.7)	0.096
CD4+/CD8 + ratio	0.83 (0.64–1.3)	0.80 (0.60–1.3)	0.90 (0.70 to 1.30)	0.01 (−0.06 to 0.08)	0.95 (−9.09 to 8.66)	0.651	0.02 (−0.06 to 0.1)	2.94 (−6.98 to 9.59)	0.290
Haemoglobin (g/L)	15.0 (14.0–15.8)	15.1 (13.9–15.9)	15.1 (14.0–15.8)	0 (−0.5 to 0.6)	0 (−2.94 to 4.2)	0.804	0 (−0.55 to 0.5)	0 (−3.98 to 3.53)	0.918
AST (UI/mL)	21.0 (18.0–26.0)	20.0 (17.7–26.0)	21.0 (18.0–26.0)	1 (−3.25 to 3.25)	−4.3 (−16.22 to 18.32)	0.651	0 (−4 to 4)	0 (−17.03 to 19.38)	0.906
ALT (UI/mL)	20.0 (15.0–27.0)	20.0 (14.0–28.0)	19.0 (14.0–27.2)	−1 (−5.25 to 4)	−6.92 (−22.22 to 22.04)	0.651	0 (−6.25 to 4.25)	0 (−23.19 to 25.42)	0.906
Glycemia (mg/dL)	94.0 (86.0–105.5)	92.0 (85.5–105.0)	90.0 (83.0–104.0)	0 (−9 to 8)	0 (−8.27 to 9.22)	0.651	−2 (−11 to 4)	−2.42 (−11.11 to 4.44)	**0.019**
Total cholesterol (mg/dL)	200 (173–220)	184 (159–204)	187 (169.3–221.8)	−13 (−26.5 to 5.25)	−6.17 (−14.24 to 2.76)	**0.001**	0 (−27 to 14)	0 (−12.86 to 8.07)	0.181
LDL cholesterol (mg/dL)	119 (97–144)	104 (80–126)	115 (94–138)	−6 (−21 to 6)	−6.67 (−17.21 to 6.96)	**0.025**	0 (−20 to 11)	0 (−18.1 to 10.47)	0.445
HDL cholesterol (mg/dL)	48 (41.2–61.5)	(42–58.7)	51 (43–60)	0 (−5 to 4.75)	0 (−10.94 to 8.97)	0.651	1 (−4 to 7)	2.56 (−8.7 to 17.02)	0.117
TC/HDL ratio	3.9 (3.3–4.8)	3.5 (3.1–4.4)	3.7 (3.1–4.6)	−0.22 (−0.56 to 0.22)	−6.33 (−16.19 to 5.81)	**0.027**	0.17 (−0.56 to 0.21)	−4.82 (−14.97 to 4.27)	**0.017**
Triglycerides (mg/dL)	122 (92.2–166.8)	107 (85.7–144.0)	112 (86.0–155.0)	−12 (−43.5 to 14.5)	−12 (−29.2 to 12.35)	**0.025**	−7 (−43 to 15)	−5.83 (−29.59 to 15.82)	**0.024**
Creatinine (mg/dL)	1.00 (0.89–1.13)	1.00 (0.86–1.12)	0.990 (0.87–1.14)	−0.01 (−0.08 to 0.07)	−0.83 (−8.03 to 7.29)	0.651	0 (−0.08 to 0.08)	0 (−7.77 to 8.42)	0.918
eGFR-EPI-CKD 2021 (ml/min/1.73m2)	80.3 (65.3–99.6)	79.7 (68.4–98.4)	79.2 (61.6–98.7)	1.5 (−6.3 to 8.1)	2.08 (−7.54 to 12.15)	0.651	−0.2 (−8.15 to 5.35)	−0.36 (−8.05 to 7.11)	0.906
IL-6 (ng/mL)	206 (158.5–356.9)	-^*b*^	165.8 (159.4–209.0)	-	-	-	−13 (−145 to 27.75)	−6.96 (−39.37 to 14.99)	**0.019**
hs-CRP (pg/mL)	97.5 (60.8–148.5)	-	73.0 (28.9–122.6)	-	-	-	−27 to 95 (−63.99 to 25.98)	−33.05 (−52.64 to 29.54)	**0.019**
D-dimer (ng/mL)	46.2 (30.2–71.2)	-	51.8 (36.1–85.2)	-	-	-	4.41 (−26.61 to 33.67)	9.97 (−49.93 to 101.73)	0.530

^*a*^ The table summarizes raw values of and changes between baseline (BL). week 24 (w24), and week 48 (w48). On the left side, the table reports the medians (first quartile–third quartile) values at the three time-points for the variables of interest. On the right side, the table reports two couples of three columns, one for each comparison (w24 versus BL and w48 versus BL). For each comparison, the table reports three columns: one for the median absolute difference between time-points (e.g., w48 –BL), another for the median percentage relative difference (MRD %) between time-points (e.g., 100x[w48 – BL]/BL]), and a third for the *P*-values coming from the paired Wilcoxon test between time-points (paired Wilcoxon test between w24 or w48 and BL). *P*-values < 0.05 are highlighted in bold. ALT: alanine aminotransferase; AST: aspartato aminotransferase; eGFR: estimated glomerular filtration rate; HDL: high-density lipoprotein; LDL; low-density lipoprotein; TC: total cholesterol; IL: interleukin; hs-CRP: high-sensitivity C-reactive protein.

^*b*^ -, absence of data.

### Metabolic profile

**Fig 2:**
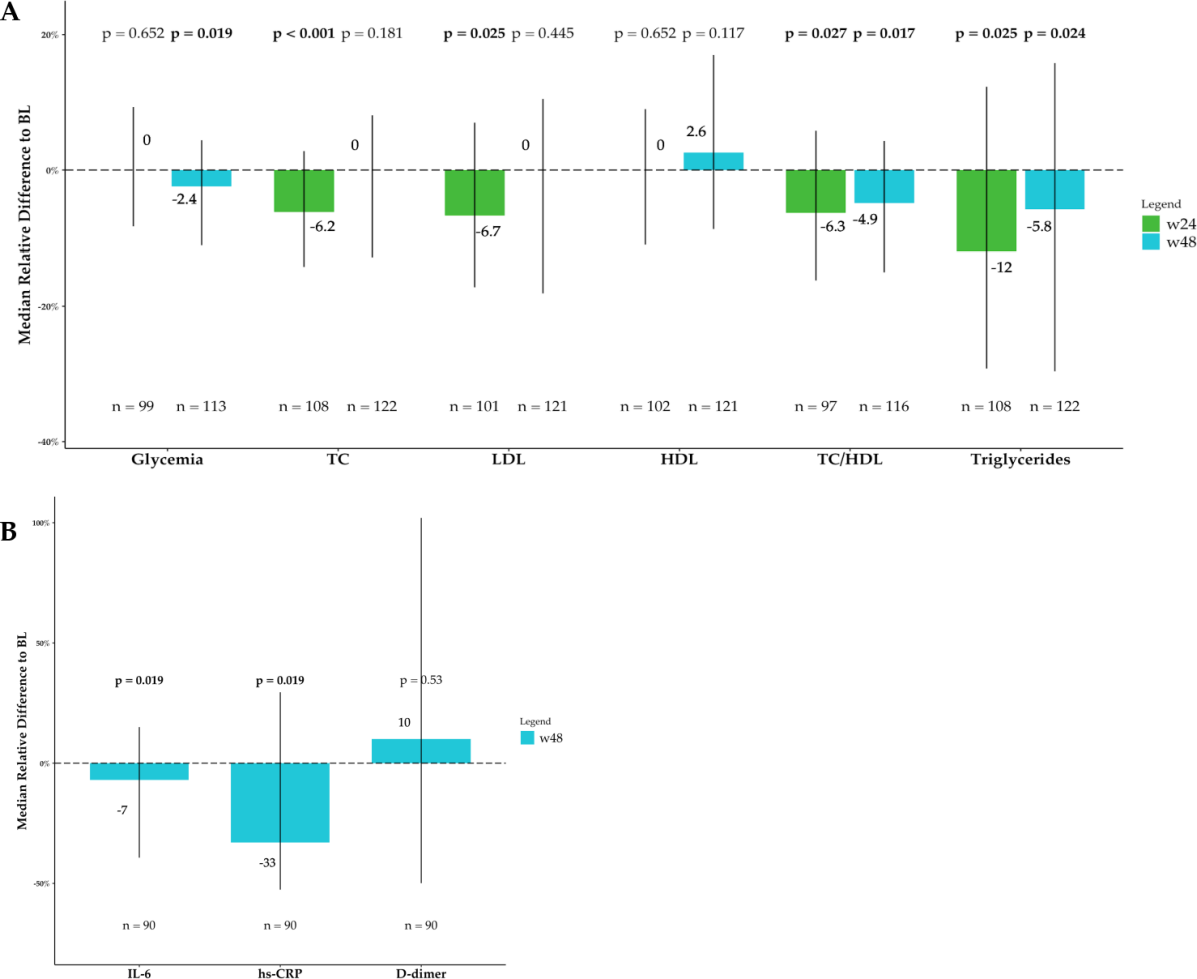
Metabolic and inflammatory changes over time. The bars show the median percentage relative difference from baseline (BL) for the metabolic parameters considered at both week 24 (w24) and week 48 (w48). The *P*-value shown upon each bar comes from the paired Wilcoxon test between BL and the considered time-point. Vertical lines show the interquartile range. (**A**) Metabolic profile. (**B**) Inflammatory profile. IL-6: interleukin 6; hs-PCR: high-sensitivity C-reactive protein; TC: total cholesterol; TC/HDL: total cholesterol/HDL cholesterol ratio.

In Table 5, the main results of the metabolic profile are reported, while the percentual changes between the different time points and the BL are depicted in Fig. 2A. Briefly, after 24 weeks of observation, the lipid profile improved, with a significant decline in triglycerides, TC, LDL, and TC/HDL ratio. These results were confirmed only for triglycerides and TC/HDL ratio at w48, when a statistically, yet not clinically significant reduction in body weight and BMI was observed. Additionally, at w48, fasting glucose levels significantly declined compared to BL. Renal and hepatic profiles did not show any significant change during the study period.

### Safety and tolerability

No treatment-related AEs attributed to the use of DOR were reported in the medical records during the follow-up. No treatment discontinuations due to tolerability issues were reported.

### Inflammatory profile

To investigate whether DOR could modulate the chronic inflammation observed in PLWH, we compared serum levels of IL-6, hs-CRP, and D-dimer, three biomarkers associated with chronic inflammation, in PLWH (16). After 48 weeks of observation, a significant decrease in IL-6 and hs-CRP was detected. D-dimer levels did not significantly differ between the study points. Results are reported in Table 5, and percentual changes from BL are shown in Fig. 2B.

## DISCUSSION

The current analysis of the DORAGE study confirms the efficacy, safety, and tolerability of the switch to a DOR-based ART in a cohort of elderly PLWH, as previously demonstrated in different populations by pivotal randomized controlled trials (9, 17) and observational studies (18, 19). Additionally, it provides novel insights into the positive impact of DOR-based regimens on chronic systemic inflammation.

DOR demonstrated high rates of virologic control in the per-protocol analysis at both w24 and w48, with no relevant virologic failure at w48: the two participants experiencing HIV-RNA >200 copies/mL showed no RAMs to DOR at GRT and eventually returned to HIV-RNA levels below 200 copies/mL by continuing the same regimen. The pharmacokinetic characteristics of DOR may explain this result: low serum protein binding, long plasmatic half-time, and its peculiar kinetic decay (4, 20, 21) all contribute to high virologic efficacy and high tolerability, with minimal percentages of drug discontinuation due to virologic failure.

International guidelines (3), as well as the summary of product characteristics (4), contraindicate DOR among PLWH with known major RAMs to DOR before the switch. Nonetheless, in the current era, which is characterized by highly efficacious and well-tolerated antiretrovirals, historical genotypic patterns that reduce DOR susceptibility are not always available. Moreover, performing GRT on HIV-DNA is not a standardized, routine practice (22), and data derived from historical GRT should be treated with caution (23). Thus, we aimed to assess whether switching to a DOR-based ART is a safe choice in the Italian context, given the prevalence of pre-switch RAMs to NNRTIs. Indeed, considering that substantial cross-resistance to DOR is detectable among NNRTI-resistant viruses harboring complex mutational patterns (24), underestimated DOR resistance could lead to virologic failure. Unlike Asiatic regions, where the prevalence of RAMs to DOR is high among NNRTI-experienced PLWH, especially among those with previous virologic failure to first-line ART (25), our data align with previous reports from Europe (18, 26–28), suggesting that the prevalence of RAMs to DOR is lower compared to other NNRTIs, both in ART-experienced PLWH and in those with previous virologic failure to NNRTIs. In the DORAGE cohort, we observed a low prevalence of pre-switch RAMs to DOR (Table 3), with a high virologic efficacy even in the presence of mutational patterns associated to resistance to DOR (Table 4).

In our cohort, several NNRTI-associated RAMs were identified prior to the switch to DOR, as shown in Table 3. Among these, the V90I and V179D mutations were observed in 3.4% and 1.1% of subjects, respectively. Importantly, these polymorphic mutations have minimal or no impact on DOR susceptibility according to the Stanford algorithm, as their drug resistance mutation score remains at 0, indicating susceptibility to DOR. Other mutations, such as A98G, V106I, and Y181C, were associated with varying levels of potential low-level resistance or low-level resistance, but they did not preclude virologic control in the subjects harboring these mutations. Therefore, while some RAMs were present, their clinical significance in terms of resistance to DOR appeared limited, as supported by the sustained virologic control observed in our cohort. Assante-Appiah *et al*. showed that occurrence of NRTI-RAMs, including thymidine analog mutations, was associated with DOR hyper-susceptibility, particularly in the absence of NNRTI RAMs (29). Whether the cumulative RT-RAM profile could improve the susceptibility to DOR in the presence of DOR-RAMs requires further investigations.

Overall, the switch to DOR was metabolically neutral, with no deterioration observed in renal or hepatic profiles. Improvements in the lipid profile were noted at w24, with a sustained benefit for the total/HDL cholesterol ratio at w48. Although a statistically significant reduction in body weight and BMI was observed at w48, this change was not clinically significant. Taken together, these findings highlight that switching to a DOR-based regimen is a safe option in terms of metabolic health, particularly among elderly PLWH, without increasing risk factors for non-AIDS events such as cardiovascular disease.

The observed improvements in metabolic profiles following the switch to a DOR-based regimen align with previous reports on the positive effects of DOR, but our cohort’s heterogeneity in both pre- and post-switch regimens makes it difficult to attribute these results solely to DOR. Participants in the DORAGE cohort had varying ART background , and post-switch regimens were equally diverse, with combinations including DOR with INSTI, and DOR with or tenofovir disoproxil (TDF) or alafenamide (TAF); moreover, some individuals switched from TAF and TDF. Despite this complexity, the longitudinal improvements in key metabolic parameters, including fasting glycemia, cholesterol, triglycerides, and body weight, remain noteworthy. The potential for DOR-based regimens to improve metabolic health, particularly in those switching from dyslipidemia-inducing therapies, remains compelling and warrants further exploration in larger cohorts.

Notably, the combination of DOR with INSTIs has emerged as a promising option, particularly in older PLWH with multiple comorbidities. Despite the fact that current international guidelines do not recommend this association (3), its use has been widely implemented in real-life experience because of several advantages in case of multidrug-resistant viral strains, drug–drug interactions with proton-pump inhibitors, renal failure, and boosted PI-related iatrogenic dyslipidemia (30–32). In this regard, Mazzitelli *et al*. showed a significant reduction in body weight and BMI in a small cohort of 18 PLWH older than 50 3 months after switching to a INSTI-DOR dual ART, but no effects on the lipidic profile (31). Moreover, a recent multicenter retrospective observational study on the switch to an INSTI + DOR dual regimen from a previous INSTI-based regimen (DORINI) showed high durability and positive metabolic properties after 12 months from the switch [32]. Overall, the existing literature and our findings suggest that DOR, whether combined with INSTIs, represents a safe and effective option for ART-experienced PLWH.

To the best of our knowledge, this is the first study to investigate the modulatory properties of DOR-based regimens on chronic systemic inflammation. After 48 weeks from the switch to DOR, we showed a significant decline in hs-CRP and IL-6 levels. The DORAGE cohort exclusively enrolled only elderly PLWH, a population burdened with a higher prevalence of comorbidities compared to the general population. The opportunity to modulate the systemic inflammatory milieu by reducing the serum concentration of pro-inflammatory biomarkers represents an intriguing finding in this population. Moreover, the general positive influence of DOR-based regimens on the metabolic profile, particularly on serum fasting lipid changes over time, clearly impacts the cardiovascular risk and its related major adverse events. Longer observational studies will answer whether these encouraging results will translate into a slowdown of the inflamm-aging process by a reduction in the incidence of non-AIDS-defining, HIV-1-related comorbidities.

The current study presents some limitations. First, our prospective analysis halts at w48: a longer observation is warranted to confirm the persistence of the results observed and the durability of the DOR-based regimens. Second, the sample size, while comparable to those in other real-life observational studies, along with the heterogeneity of the pre- and post-switch regimens and the absence of a control arm, limits the ability to draw definitive conclusions about the performance of different antiretroviral regimens: larger sample sizes and clearer study designs are needed for more robust statistical analyses in this regard. Third, the lack of a BL assessment with repeated measurements before the switch, especially for the evaluation of the inflammatory biomarkers, does not account for intra-individual variability over time.

In conclusion, DOR-based regimens are effective, safe, and well-tolerated among elderly PLWH. Pretreatment DOR-RAMs are uncommon in the Italian context among ART-experienced PLWH, and DOR showed virologic efficacy even in the presence of multidrug-resistant viral strains. After 48 weeks from switching from any ART, a favorable improvement in fasting glycemia and lipidic profile was recorded, along with a neutral impact on immune, hepatic, and renal profiles. An intriguing modulatory effect of DOR-based regimens on chronic systemic inflammation was detected. Larger studies are needed to confirm our results and extend them to key populations generally underrepresented in pivotal randomized controlled trials.
